# Neutral endopeptidase (NEP) is differentially involved in biological activities and cell signaling of colon cancer cell lines derived from various stages of tumor development

**DOI:** 10.1007/s13277-016-5248-y

**Published:** 2016-07-27

**Authors:** Magdalena Mizerska-Kowalska, Agnieszka Bojarska-Junak, Joanna Jakubowicz-Gil, Martyna Kandefer-Szerszeń

**Affiliations:** 1 Department of Virology and Immunology, Maria Curie-Sklodowska University, Faculty of Biology and Biotechnology, Akademicka 19 Street, 20-033 Lublin, Poland; 2 Chair and Department of Clinical Immunology, Medical University of Lublin, W.Chodźki 4a Street, Lublin, Poland; 3 Department of Comparative Anatomy and Anthropology, Maria Curie-Sklodowska University, Faculty of Biology and Biotechnology, Akademicka 19 Street, 20-033 Lublin, Poland

**Keywords:** NEP, Neutral endopeptidase, Colon cancer, Akt/PTEN, FAK, Apoptosis, Cell cycle

## Abstract

The presented studies were aimed at exploring the role of neutral endopeptidase (NEP) in the function of colon cancer cell lines LS 180 and SW 620, derived from different grades and stages of tumor development. NEP silencing by siRNA resulted in decreased viability and proliferation accompanied by increased apoptosis in both cell lines. Additionally, cell cycle arrest at the G2/M phase was observed, but only in LS 180 cells. Opposite to these results, serum-stimulated migration was increased in both cell lines. Furthermore, NEP silencing influenced the invasive activity of LS 180 and SW 620 cells in an opposite manner: while LS 180 cells showed an enhanced invasiveness, SW 620 cells exerted a reduced activity. An exploration of the activity of signaling molecules responsible for the function of tumor cells—Akt, PTEN, and FAK—after NEP silencing indicated that the endopeptidase is involved in their regulation. The increased phosphorylation level of Akt was accompanied by a decrease in PTEN in the presence of a high concentration of serum. A reduced concentration of serum did not change the phosphorylation status of Akt. Enhanced autophosphorylation of FAK was observed in LS 180 and SW 620 cells cultivated in a medium with a high concentration of serum. Taken together, these results confirm that NEP is implicated in the regulation of the survival, growth, and motile activity of colon cancer. This is also the first report which shows that NEP mediates cancer cell migration and invasiveness, but not growth and survival, through Akt/FAK signaling pathways.

## Introduction

In tumor cells, survival and proliferation, inhibition of apoptosis, invasion and migration, as well as the induction of angiogenesis during carcinogenesis, are regulated by autocrine and paracrine growth factors, cytokines, hormones, and signaling peptides. The availability of these extracellular signaling molecules is regulated through proteolysis, which may be mediated among others by cell membrane-bound peptidases, expressed on the surface of tumor and stromal cells. One of such peptidases is neutral endopeptidase (NEP), also called neprilysin, enkephalinase, CD10, EC 3.4.24.11, and common acute lymphoblastic leukemia antigen (CALLA) [1, 2]. NEP could be a very useful tool for the diagnosis and prognosis of B-lineage acute lymphoblastic leukemia and several carcinomas originating from the kidney, lung, skin, pancreas, prostate, liver, breast, stomach, cervix, and bladder. It has been determined that NEP can be up or downregulated in neoplastic cells. Moreover, it should be underlined that the expression level of NEP is dependent on the proliferation and differentiation status of tumor cells. NEP is implicated both indirectly and directly in the regulation of signaling pathways. Its indirect action involves proteolytic degradation or activation of bioactive peptides, growth factors, and cytokines. In addition to this, NEP regulates signaling pathways in a direct fashion. This peptidase acts as a receptor anchored in the membrane through GPI complexes. Studies have shown that CD10 (NEP) is implicated in cell migration, cell proliferation, and survival through FAK and PTEN/Akt signaling pathways in prostate cancer (PC) [1–6].

The phosphatase and tensin homolog detected on chromosome ten phosphatidylinositol-3,4,5-trisphosphate 3-phosphatase (PTEN) acts as a tumor suppressor by antagonizing the activity of PI-3 kinase. PTEN dephosphorylates the secondary messenger phosphatidylinositol 3,4,5-triposphate (PIP_3_). This, in turn, results in the downregulation of the Akt kinase activity. Akt is a pivotal molecule in the oncogenic pathway which promotes cell growth, survival, and invasiveness [7]. PTEN is frequently inactivated in different manners in a number of cancers, which results in an increased activity of Akt and tumor progression [8, 9]. It should be underlined that PTEN exerts its tumor-suppressive activity also by regulating cell cycle and cell differentiation [10, 11].

Focal adhesion kinase (FAK) is a tyrosine kinase whose activity is tightly connected with cell adhesion to the extracellular matrix through integrin receptors. The regulation of FAKs influences cellular events that are either dependent on cell adhesion, such as cell proliferation and survival, or that require modulation of cell adhesion, such as cell migration. Involvement of FAKs in the cellular pathways which regulate cell growth and cell movement implicates it in the development of cancer and other diseases [12].

Colon carcinoma (CC) is one of the most prevalent tumors in the world. The life expectancy of patients with a metastatic form of this tumor has been extended in the past decade, but the disease still remains incurable. The development of CC, which starts with changes in a normal colonic epithelium through adenomatous polyps to metastatic cancer, is dependent on and supported by the tumor microenvironment. In the case of CC, information about the role of tumor-associated NEP is rather scanty. There are reports which indicate that this peptidase contributes to liver metastasis of CC cells by degrading the antitumoral peptide, Met-enkephalin [13, 14]. Most studies consider the expression levels of NEP proteins in the tissue samples of colon adenocarcinoma in comparison with adjacent non-neoplastic tissues and with respect to the degree of tumor differentiation, tumor development, progression to more advanced stages, invasion, and metastasis. The studies are discrepant in some aspects. De Oliveira et al. and Sato et al., for instance, have reported that the level of NEP expression was higher in CC tissue samples than in non-neoplastic mucosa adjacent to the tumor, whereas Ogawa et al. did not find any expression of this marker in samples of normal tissue [15–17]. Sato et al. and Fujimoto et al. identified a higher expression level of NEP in well or/and moderately differentiated adenocarcinoma tissue specimens in comparison with poorly differentiated ones [16, 18]. On the other hand, de Oliveira et al. and Oshima et al. did not observe any differences in NEP expression corresponding to histological differentiation [15, 19]. Lymphatic, vascular, and/or perineural invasion of colorectal carcinoma was associated with NEP expression according to Fujimoto et al. and Yao et al., whereas de Oliveira et al. and Fujita et al. did not observe any significant relationships in this respect [15, 18, 20, 21]. Moreover, there are some controversies regarding the expression level of the peptidase and metastasis. Fujimoto et al. and Yao et al. have identified a strong expression of this marker in patients with a higher incidence of liver metastases [18, 21]. On the other hand, de Oliveira et al. and Fujita et al. did not find any such relationship [15, 20].

In the present study, we examined the role of NEP in the function of CC cells derived from different grades (II and IV) and stages (B and C according to Duke’s classification) of tumor development and presenting high levels of NEP expression. Moreover, we investigated the role of NEP in the regulation of Akt/PTEN and FAK signaling pathways.

## Material and methods

### Cell cultures

The human colon cancer cell lines obtained from the American Type Culture Collection (ATCC), HT-29 (no. HTB-38), LS 180 (no. CL-1870), SW 948 (no. CCL-237), and SW 620 (no. CCL-227), were used in these studies. These cell lines represented different grades and stages of cancer development (Duke’s classification): HT-29 (Duke’s stage A, grade I), LS 180 (Duke’s stage B, grade II), SW 948 (Duke’s stage C, grade III), and SW 620 derived from a lymph node metastatic site of tumor (Duke’s stage C, grade IV). They were maintained in appropriate media recommended by ATCC, namely, HT-29 in McCoy’s 5A medium, LS180 in MEM, and SW948 and SW620 in Leibovitz’s L-15 medium. All media were supplemented with fetal calf serum (FCS) at different concentrations, depending on study requirements, plus 100 U/ml penicillin and 100 μg/ml streptomycin. Cell cultures were cultivated under standard conditions at 37 °C, 95 % humidity and with (HT-29 and LS 180) or without (SW 948 and SW 620) 5 % CO_2_.

### *NEP* gene expression silencing

Inhibition of *NEP* gene expression was achieved by RNA interference with short interfering RNA (siRNA) against human *NEP*. Silencer® Select siRNA targeted to the *NEP* gene (siNEP) and negative control siRNA (siCtrl) were purchased from Invitrogen™ (Thermo Fisher Scientific). At first, three different siRNAs were tested to determine which of them provide the highest level of NEP silencing. Ultimately, the sense sequence of siNEP CGGCUAUCCUGAUGACAUUtt and the antisense sequence AAUGUCAUCAGGAUAGCCGat were used in studies. A non-siRNA control (cells not treated with siRNA) was also included in these studies. Depending on assay requirements, transfection was performed in 6-well plates, T25 flasks, or Lab-Tek™ microscope slides. In these experiments, two-step transfection (reverse transfection followed by forward transfection) was used which allowed us to achieve the best level of NEP silencing. As the first step of silencing, we diluted siRNA in Opti-MEM® I medium in cell cultureware. Then, Lipofectamine™ RNAiMax was added. The mixture was incubated for 20 min at room temperature (RT). Next, LS 180 and SW 620 cells were suspended in a culture medium supplemented with FCS without antibiotics. The LS 180 cell line was used at a density of 7.5 × 10^4^ cells/ml and SW 620 at 8.5 × 10^4^ cells/ml. Cells were added to complexes of siRNA and Lipofectamine™ RNAiMax, mixed gently, and then incubated for 24 h under standard conditions. After that, forward transfection was conducted. In this step, siRNA and Lipofectamine™ RNAiMax were diluted in Opti-MEM® I medium separately, mixed gently, and incubated for 5 min at RT. Next, they were combined, mixed, and incubated for 20 min at RT. Complexes of siRNA and Lipofectamine™ RNAiMax were added to cells and incubated for 24 h. After this, colon cancer cells were subjected to further assays. In each step of transfection, siRNA was used at 10 nM of final concentration combined at a 1:1 volume ratio with a lipid carrier. The effectiveness of gene silencing was determined by immunofluorescence staining and flow cytometry as described below.

### Immunofluorescence detection of NEP

Immunofluorescence staining was performed to determine the presence of NEP in the colon cancer cell lines. For this purpose, cells were cultivated on Lab-Tek™ microscope slides (Chamber Slide™ Systems, Thermo Scientific) for 48 h under standard conditions and in the presence of 10 % FCS. Afterward, cells were washed three times with PBS and fixed for 10 min in 3.7 % paraformaldehyde in PBS. After washing, cells were treated with 0.2 % Triton X-100 for 7 min and once again washed with PBS. Subsequently, a blocking step in 5 % goat serum was performed for 30 min at RT. Cells were then incubated with mouse antihuman NEP mAb (Santa Cruz Biotechnology, Inc.) (1:250), washed with PBS, and incubated with goat anti-mouse IgG-FITC secondary antibodies (Santa Cruz Biotechnology Inc.) (1:500). Labeled cells were mounted in UltraCruz® Mounting Medium (Santa Cruz Biotechnology Inc.) containing DAPI stain and examined under the LSM 5 Pascal/AxioVert 200M confocal microscope (Carl Zeiss). Negative control comprised cells incubated with secondary antibodies alone.

### Flow cytometry analysis of NEP expression

Fluorescence-activated cell sorting (FACS) was performed to quantify the level of NEP in the colon cancer cell lines HT-29, LS 180, SW 948, and SW 620. For this purpose, cells were cultivated in six-well plates in the presence of 10 % FCS for 48 h under standard conditions. The cells were detached with Accutase® Solution, resuspended in a medium with 1 % FCS, and incubated for 60 min under standard conditions. Afterward, cells were centrifuged at 300×*g* for 5 min and washed with PBS. Then, cells were incubated with phycoerythrin (PE)-conjugated mouse antihuman NEP mAb IgG (BD Biosciences) for 30 min at RT in darkness. In addition to this procedure, a permeabilization step was also incorporated to enable intracellular staining of the cells. After washing with PBS, FACS data, acquired by running the samples on a FACS Calibur (BD Biosciences), were analyzed using Cell Quest software (BD Biosciences). The data were expressed as percent of cells expressing NEP and relative mean fluorescence intensity (MFI). IgG isotype control Ab (BD Biosciences) was used as a negative control.

### In vitro viability assay

The viability of LS 180 and SW 620 colon cancer cells after NEP silencing was determined by the Neutral Red uptake assay (Tox-4, Sigma). For this purpose, cells cultivated in T25 flasks were transfected as described above. After that, the cells were harvested and plated in 96-well plates. For the viability assay, 5.5 × 10^5^ cells/ml (LS 180) and 8.5 × 10^5^ cells/ml (SW 620) were used. After the cells adhered, the cell culture medium was changed to one supplemented with 2 % (restricted proliferation of the cells) FCS. The colon cancer cells were incubated for 48 h. Afterward, the Neutral Red uptake assay was performed according to the manufacturer’s instruction. The viability of the cells was expressed as percent of the non-siRNA control (100 % of living cells).

### In vitro proliferation assay

The proliferation activity of LS 180 and SW 620 cells after NEP silencing was established using a method based on the incorporation of bromodeoxyuridine (BrdU) into the DNA of actively proliferating cells and detection of the nucleotides by specific antibodies coupled with an enzyme (ELISA) (Roche Molecular Biochemicals). Transfection was performed in six-well plates. After harvesting, the cells were plated in 96-well plates at a density of 5.5 × 10^4^ cells/ml (LS180) and 8.5 × 10^4^ cells/ml (SW 620). Cells were cultivated in an appropriate medium supplemented with 10 % (extensively proliferating cells) FCS for 72 h. Additionally, in some experiments, IL-8 (Cell Signaling Technology, Inc.) was added to the culture medium at a final concentration of 10 ng/ml. After incubation, the BrdU assay was performed according to the manufacturer’s instruction. The optical density was measured at 570 nm by means of a Universal Plate Reader, EL800 (Bio-Tek Instruments, Inc.). The proliferation activity of the colon cancer cells after NEP silencing was expressed as percent of the non-siRNA control (100 % of actively proliferating cells).

### In vitro migration and invasion assays

The migration and invasiveness assays were performed in transwells. The migration assay was conducted in transwells equipped with membrane inserts (pore size, 8 μm) in 24-well plates. The invasiveness of the colon cancer cells was assessed based on the number of cells invading through the basement membrane extract (BME)-coated membrane (8 μm) of transwell chambers using the Trevigen’s CultureCoat® 96-well Cell Invasion Assay (R&D Systems). The assay was performed according to the manufacturer’s instruction with a minor modification. Prior to the assays, colon cancer cells were starved in a medium supplemented with 0.5 % FCS overnight. After that, LS 180 and SW 620 cells were harvested and suspended in a medium with 2 % FCS to a density of 8.5 × 10^5^ and 1.0 × 10^6^ cells/ml, respectively. Cells were added to the top chambers. Bottom chambers were filled with an appropriate medium supplemented with 2 % FCS (no chemoattractant gradient—spontaneous migration/invasion) or 10 % FCS (a chemoattractant gradient—chemoattracted migration/invasion). After 72 h of incubation under standard conditions, the number of colon cancer cells invading to the lower surface of the insert membrane was quantified using Calcein AM, which, after internalization into the cells, is processed to free calcein. Free calcein fluoresces brightly. The fluorescence was measured by means of a Perkin Elmer Victor™ plate reader. The fluorescence obtained in the non-siRNA control corresponded to 100 % of migration or invasion activity. The results were expressed as percent of the non-siRNA control.

### Apoptosis analysis

FACS along with a modified annexin V/propidium iodide protocol (FITC AnnexinV Apoptosis Detection Kit II, BD Pharmingen™) were used to determine apoptosis (as percent of total cell number) in colon cancer cells after NEP silencing. Briefly, cells were cultivated in six-well plates in the presence of 2 or 10 % FCS for 72 h under standard conditions after transfection. After harvesting and suspending in annexin V binding buffer, cells were incubated with annexin V and subsequently with PI. After that, cells were permeabilized with formaldehyde to promote entry of RNase into the cells following staining. The addition of RNase (Sigma) limits the number of false-positive events resulting from PI staining of RNA within the cytoplasm. FACS data were acquired on a FACS Calibur (BD Biosciences) and analyzed using Cell Quest software (BD Biosciences). The following controls were used: unstained cells and cells stained with FITC Annexin V or PI only.

### Cell cycle analysis

LS 180 and SW 620 cells growing in six-well plates were transfected as described above. After transfection, colon cancer cells were cultivated in a medium with 2 or 10 % FCS for 72 hours. Next, cells were harvested, washed with PBS, and fixed overnight with 80 % ethanol at −20 °C. After that, cells were washed with PBS and stained with a solution containing PI and RNase for 30 min in darkness at RT. The cells were analyzed by means of FACS Calibur (BD Biosciences) and FACS data were processed using Cell Quest software (BD Biosciences). The results were presented as percentage of cells in phases G_0_/G_1_, S, and G_2_/M.

### Western blot analysis

To extract total proteins, colon cancer cells were rinsed with ice-cold PBS and lysed with RIPA buffer (Sigma) supplemented with a phosphatase inhibitor cocktail (Sigma) and a protease inhibitor cocktail (Sigma) for 30 min on ice. Cells were collected and centrifuged for 10 min at 14,000 rpm and 4 °C. Supernatants were collected, and total protein concentration was determined by means of a method based on bicinchoninic acid (Pierce® BCA Protein Assay Kit, Thermo Scientific). Samples containing 50 μg were denatured and separated by 10 % SDS-PAGE gel electrophoresis (Bio-Rad, Mini-Protean® Tetra Cell). After that, proteins were transferred (Bio-Rad, Transfer-Blot® SD Semi-Dry Transfer Cell) to a PVDF membrane (Millipore, Immubilom-P). The membrane was incubated with blocking buffer (5 % non-fat dry milk or BSA) in Tris-buffered saline (TBST) with 0.1 % Tween-20, depending on the recommendation of the producer of the primary antibodies used. After blocking, the membranes were incubated with primary antibodies diluted in TBST overnight at 4 °C. The primary antibodies were rabbit anti-total FAK and anti-phospho (Tyr397 and 925) FAK, rabbit anti-total PTEN and anti-phospho (Ser380/Thr382/383) PTEN, and rabbit anti-total Akt and anti-phospho (Thr308) Akt (1:1000) (Cell Signaling Technology Inc.). To detect phosphospecific antibodies, 0.01 % of the phosphatase inhibitor cocktail was added to the blocking buffer and the antibody diluent. Afterward, the membranes were washed with TBST three times and subjected to HRP-conjugated secondary antibodies (goat anti-rabbit, Amersham™ GE Healthcare) for 1 h at RT. Protein-antibody complexes were visualized with the ECL™ Western Blotting Analysis System (Amersham™ GE Healthcare) and Molecular Imager® ChemiDoc™ XRS^+^ equipped with ImageLab™ software. The blots used to detect the phosphorylated form of proteins were stripped and reprobed with respective anti-total protein antibodies (Santa Cruz Biotechnology Inc.) as described above. β-actin (anti-β-actin antibodies, Santa Cruz Biotechnology Inc., 1:500) was also used as a load control. Additionally, protein molecular markers (Precision Plus Protein™ Dual Color Standards) were loaded onto electrophoretic gels to control the molecular weight of the protein bands.

## Results

### Differential expression of NEP in colon cancer cell lines

**Fig. 1:**
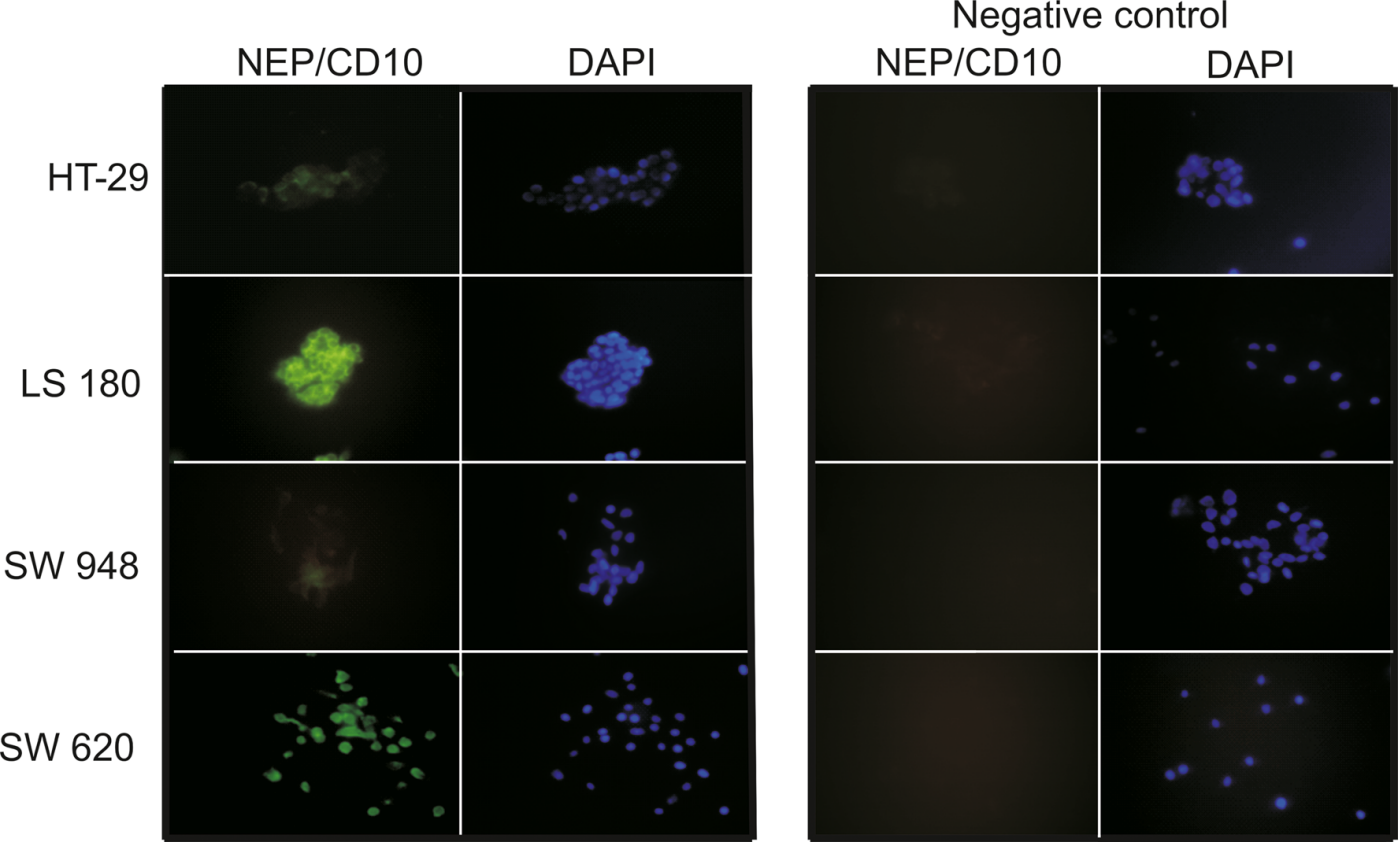
Immunofluorescence staining of NEP in the indicated colon cancer cell lines. Cells were indirectly labeled using mouse anti-human NEP antibodies and FITC-conjugated goat anti-mouse IgG-FITC secondary antibodies. The nuclei were stained with DAPI. Negative control comprised cells incubated with secondary antibodies alone and counterstained with DAPI. Representative photographs are shown in the figure

**Fig. 2:**
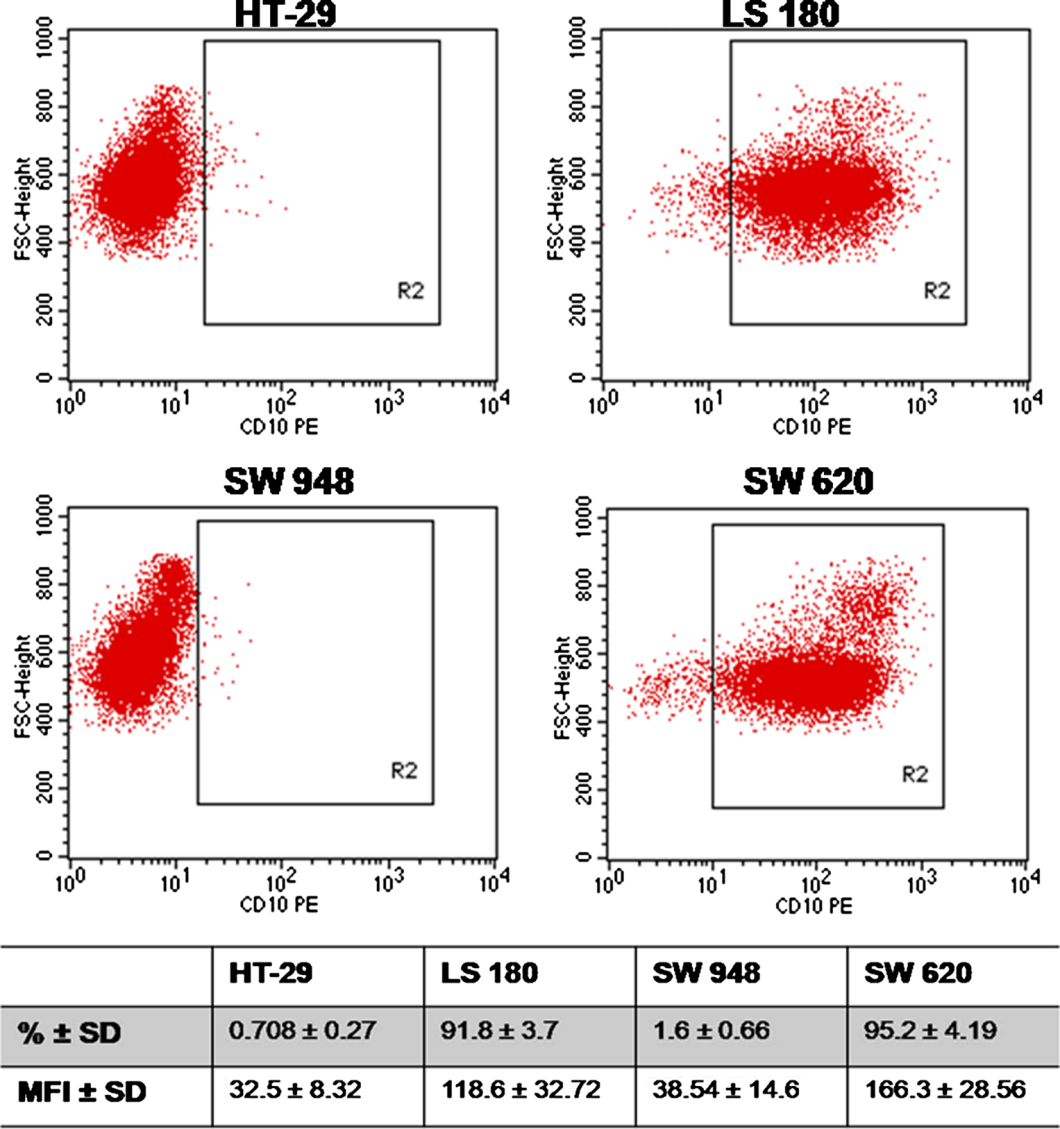
The expression profiles of NEP in the HT-29, LS 180, SW 948, and SW 620 cell lines characterized by flow cytometry. Cells were labeled with PE-conjugated mouse anti-human NEP antibodies. R2 gates in dot plots included NEP-positive cells. The table presents the percent (%) of NEP-positive cells and mean fluorescence intensity (MFI). The results are mean values (±SD) of four independent experiments

The expression of NEP in colon cancer lines was established by immunofluorescence staining and flow cytometry. Microscopic analysis revealed that colon cancer cells exhibited different patterns of NEP expression. LS 180 and SW 620 cell lines showed the most pronounced fluorescence (Fig. 1). As can be seen in the confocal microscopy images, NEP was located peripherally. The flow cytometry analysis demonstrated that SW620 cells, which are metastatic colon cancer cells (Duke’s stage C; grade IV) present in lymph nodes, showed the highest level of NEP expression (92.5 % ± 4.9; MFI 166 ± 28.56) (Fig. 2). However, LS 180 cells, representing stage B of colon cancer according to Duke’s classification (grade II), also strongly expressed NEP, as detected by flow cytometry (91.8 % ± 3.7; MFI 118.6 ± 32.72). Additionally, it was observed that in permeabilized cells before NEP detection, the MFI value was higher by approximately 20 % in both cell lines (data not presented).

### NEP is involved in the regulation of proliferation and metastatic activity of colon cancer cells

**Fig. 3:**
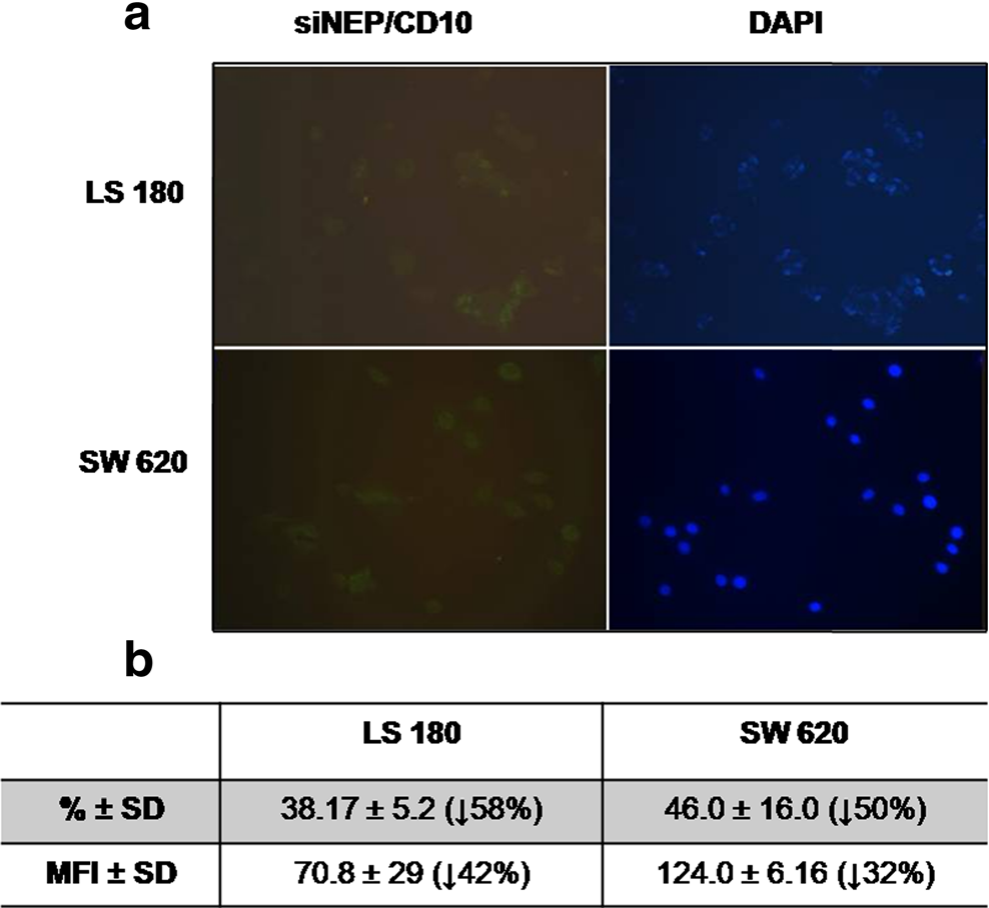
A decrease in NEP expression in the LS 180 and SW 620 cells after transfection with siRNA against human NEP. **a** Immunofluorescence staining of NEP. Cells were stained as indicated in Fig. 1. **b** The table presents the percent (%) of NEP-positive cells and MFI estimated by flow cytometry. The results are mean values (±SD) of all independent analyses carried out

**Fig. 4:**
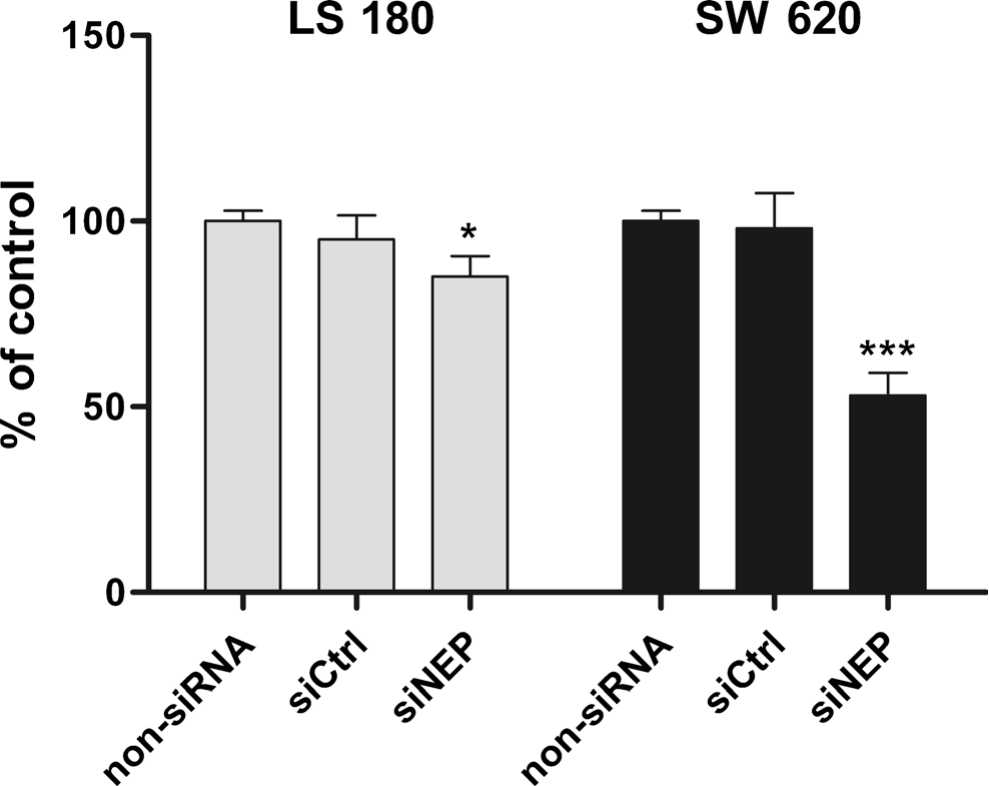
A decrease in the viability of LS 180 and SW 620 cells 48 h after NEP silencing, as determined by the Neutral Red uptake assay. The results were expressed as percent of a non-siRNA control (100 % of viable cells). The results are mean values (±SD) of three independent experiments. Differences were considered statistically significant at *p* < 0.05 (Student’s *t* test) in comparison with non-siRNA and siCtrl

**Fig. 5:**
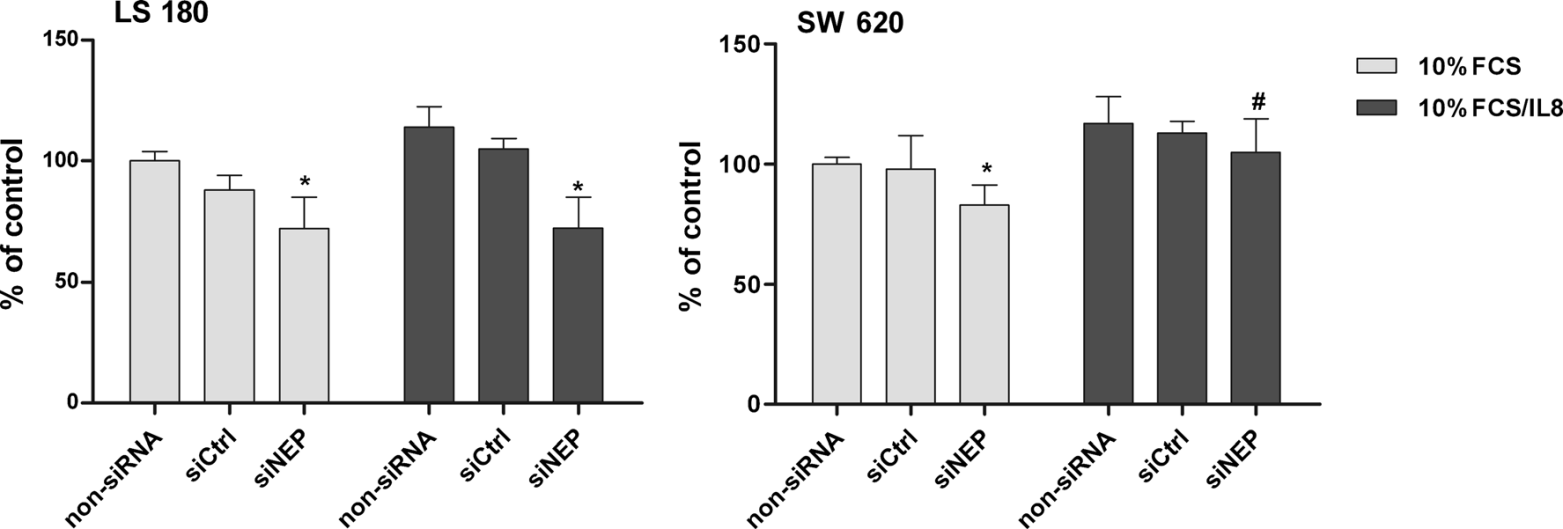
Inhibition of the proliferation of LS 180 and SW 620 cells determined by the BrdU method 72 h after NEP silencing. Cell cultures were cultivated in the presence of 10 % FCS alone or 10 % FCS plus IL-8 (10 ng/ml). The results were expressed as percent of a non-siRNA control (100 % of actively proliferating cells). The results are mean values (±SD) of three independent experiments. Differences were considered statistically significant at *p* < 0.05 (Student’s *t* test) in comparison with appropriate non-siRNA and siCtrl (*) or siNEP/10 % FCS (#)

The function of NEP in the biology of colon cancer cells was investigated in an experiment with LS 180 and SW 620 cell lines, which had the highest levels of NEP expression. By silencing NEP expression with specific siRNA, we were able to examine the role of NEP as both a peptidase capable of degrading biological active peptides and an immune receptor anchored in the membrane through GPI complexes. In the preliminary studies, three different siRNAs were tested to achieve the highest level of NEP silencing (data not presented). Ultimately, the sense sequence of siNEP CGGCUAUCCUGAUGACAUUtt and the antisense sequence AAUGUCAUCAGGAUAGCCGat were used in studies. Immunofluorescence staining showed that siNEP caused NEP silencing (Fig. 3a). Moreover, flow cytometry indicated that the number of LS 180 cells and SW 620 cells with silenced NEP was reduced by about 58 % (MFI was reduced by 42 %) and 50 % (MFI, 32 %), respectively (Fig. 3b). Silenced expression of NEP resulted in a statistically significantly decreased viability of both cell lines expressing high levels of neutral endopeptidase, namely, LS 180 and SW 620. A more pronounced decrease was observed in the case of the metastatic cell line SW 620 (by 47 %) compared with LS 180 (by 15 %) (Fig. 4). When the influence of silencing of CD10 expression was measured as proliferation activity, a decrease was also observed. There were no differences in the proliferation activity between the two types of cells as measured by the BrdU assay (Fig. 5). All the results were statistically significant in comparison with the non-siRNA and siCtrl controls. Moreover, significant differences were observed between the two cell lines when IL-8 was added to the medium as an additional growth factor. Silencing of CD10 in the LS 180 cell line decreased its proliferation activity stimulated by the growth factor. In contrast, silencing of neutral endopeptidase in SW 620 decreased spontaneous proliferation but did not affect proliferation additionally stimulated by the growth factor (Fig. 5).

**Fig. 6:**
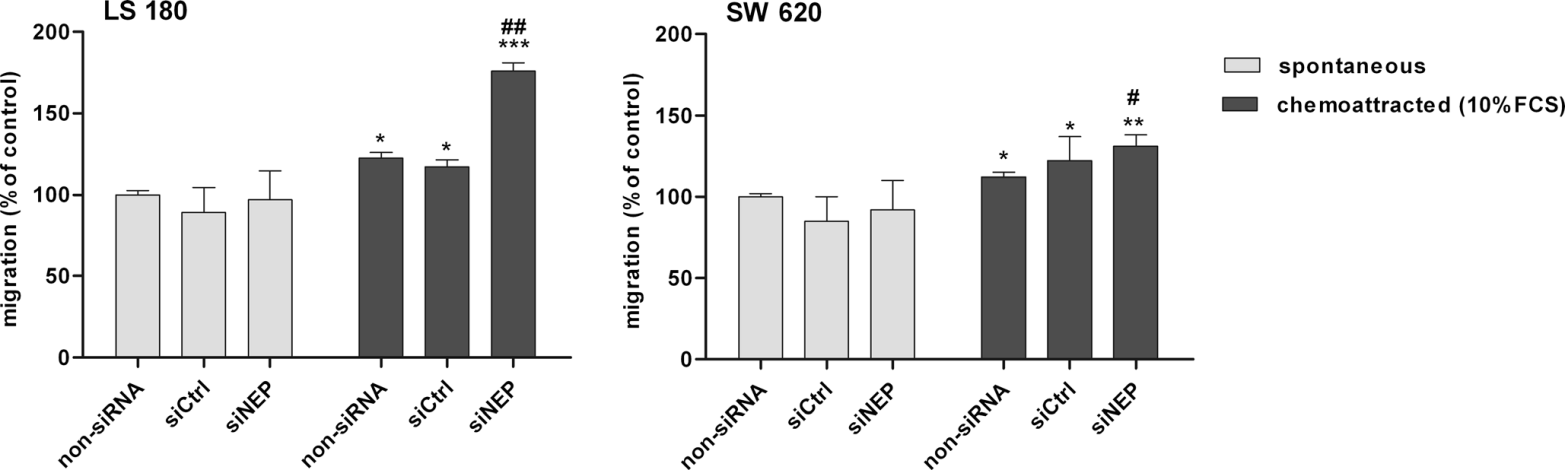
Changes in the migration activity of LS 180 and SW 620 cells detected by the transwell method. Cells migrated spontaneously or toward 10 % FCS, 72 h after NEP silencing. Colon cancer cells migrated to the lower surface of transwell membranes were quantified by measuring calcein AM fluorescence. The results were expressed as percent of migrated cells in comparison with a non-siRNA control. The results are mean values (±SD) of three independent experiments. Differences were considered statistically significant at *p* < 0.05 (Student’s *t* test) in comparison with (*asterisk*) respective non-siRNA, siCtrl, or siNEP (spontaneous) and (*number sign*) non-siRNA and siCtrl (chemoattracted)

**Fig. 7:**
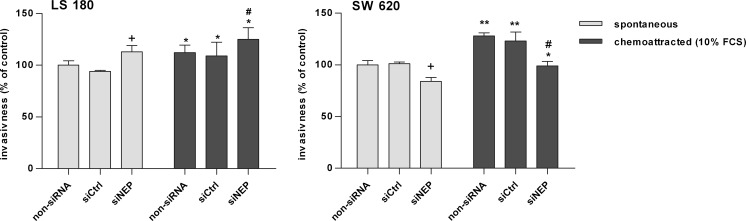
Changes in invasion of the LS 180 and SW 620 cells detected in a transwell chamber assay using a basement membrane extract (BME)-coated membrane (8 μm). Cells invaded spontaneously or toward 10 % FCS, 72 h after NEP silencing. Colon cancer cells which invaded to the lower surface of the transwell membranes were quantified by measuring calcein AM fluorescence. The results were expressed as percent of invaded cells in comparison with a non-siRNA control. The results are mean values (±SD) of three independent experiments. Differences were considered statistically significant at *p* < 0.05 (Student’s *t* test) in comparison with (*asterisk*) respective non-siRNA, siCtrl, or siNEP (spontaneous); (*number sign*) non-siRNA and siCtrl (chemoattracted) and ±non-siRNA and siCtrl (spontaneous)

In further experiments, the involvement of NEP in the migration and invasive activity of LS 180 and SW 620 cell lines was examined. As indicated in Fig. 6, silencing of CD10 by siRNA did not influence spontaneous migration of either cell line. On the other hand, enhanced serum-stimulated migration was observed. It was especially pronounced in the LS 180 cell line, which showed an about 50 % increase in migration activity in comparison to a corresponding non-siRNA control. To compare, the migration activity of SW 620 increased by only 20 %. These results were statistically significant. By contrast, measurement of invasion activity using transwells coated with BME indicated that silencing of NEP enhanced spontaneous and serum-stimulated invasive activity only in the LS 180 cell line (Fig. 7). Both spontaneous and stimulated activity were increased by approximately 13 % in comparison to an appropriate non-siRNA control. In contrast to that, in the SW 620 cell line, NEP silencing with siRNA strongly inhibited spontaneous invasive activity (about 16 %) as well as serum-stimulated activity (about 30 %) (Fig. 7). Both, the increase and the decrease observed in the in vitro invasion assay were statistically significant in comparison with appropriate controls, as indicated in the figure.

### Silencing of NEP induces apoptosis and arrest of colon cancer cells in the G2/M phase

**Fig. 8:**
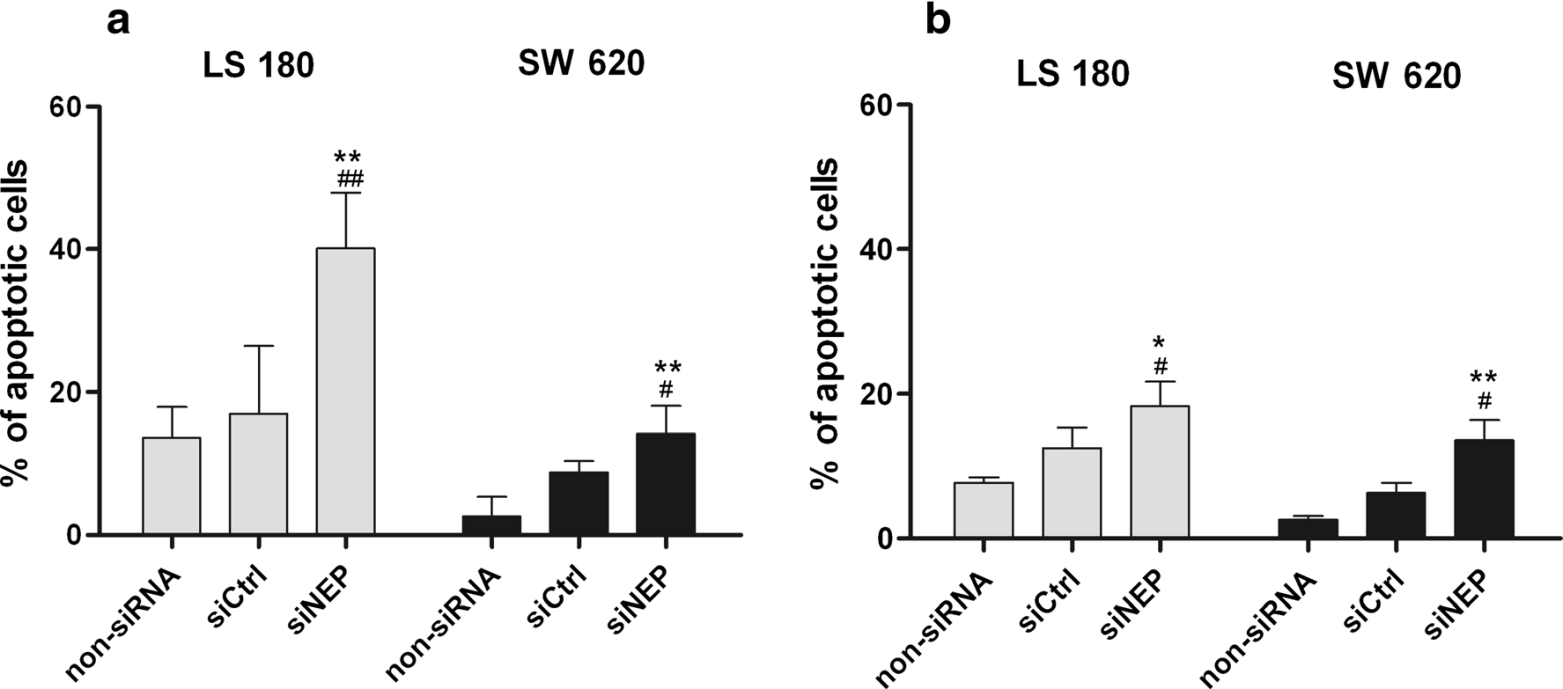
Induction of apoptosis in the LS 180 and SW 620 cell cultures after NEP silencing. Apoptotic cells (%) were detected by flow cytometry and cell labeling with Annexin V/propidium iodide 72 h after transfection. Cell cultures were cultivated in a medium supplemented with 2 % FCS (**a**) or 10 % (**b**). The results are mean values (±SD) of three independent experiments. Differences were considered statistically significant at *p* < 0.05 (Student’s *t* test) in comparison with (*asterisk*) non-siRNA and (*number sign*) siCtrl. *Black arrows* indicate bands corresponding to pFAK

**Fig. 9:**
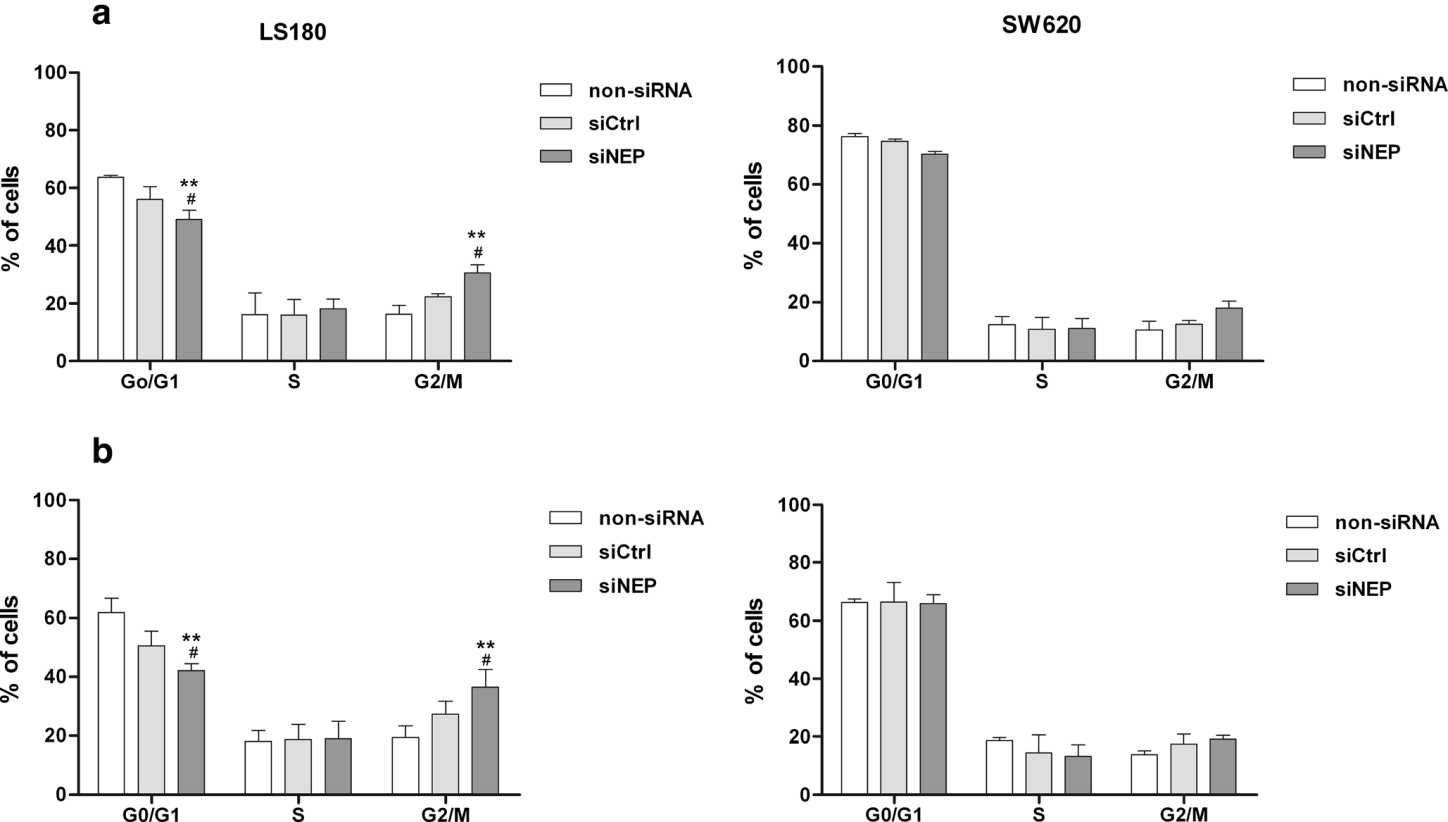
The effect of NEP silencing on cell cycle distribution in the LS 180 and SW 620 cell cultures. Cells were cultured in a medium supplemented with 2 % FCS (**a**) or 10 % (**b**) 72 h after transfection. Cell cycle distribution was determined by flow cytometry and cell staining with propidium iodide. The results are presented as percent of cells in phase G_0_/G_1_, S, and G_2_/M and mean values (±SD) of three independent experiments. Differences were considered statistically significant at *p* < 0.05 (Student’s *t* test) in comparison with (*asterisk*) non-siRNA and (*number sign*) siCtrl

As can be seen in Figs. 4 and 5, the viability and proliferation of LS 180 and SW 620 were significantly reduced by silencing of NEP. We used flow cytometry to determine whether cell death induction and/or an alteration in cell cycle may have been responsible for these outcomes. The viability and proliferation assays were carried out with medium supplemented with 2 % (restricted proliferation of the cells) and 10 % (extensively proliferating cells) FCS, respectively. The apoptosis and cell cycle analysis were also done at the same manner, to reflect experiment conditions of viability and proliferation assays. We detected that RNAi led to apoptotic, but not to necrotic cell death, in both cell lines. The level of necrosis was up to 4 % in each sample (data not presented). NEP silencing in LS 180 cells resulted in the presence of 40 and 18 % of apoptotic cells in cell cultures cultivated with 2 and 10 % FCS, respectively. SW 620 cell cultures contained about 14 % of apoptotic cells independent of the concentration of serum (Fig. 8). The increase in the number of apoptotic cells was statistically significant in comparison with non-siRNA and siCtrl. A flow cytometry analysis revealed that silencing of NEP caused a cell cycle alteration in the LS 180 cell line but not in SW 620. The former showed a significant decrease in the number of cells in the G_0/_G_1_ phase. This was accompanied by an increase in the number of LS 180 cells in the G_2_/M phase (about 16 % in comparison to a non-siRNA control) (Fig. 9).

### NEP is engaged in signal transduction via the Akt/PTEN and FAK pathways

**Fig. 10:**
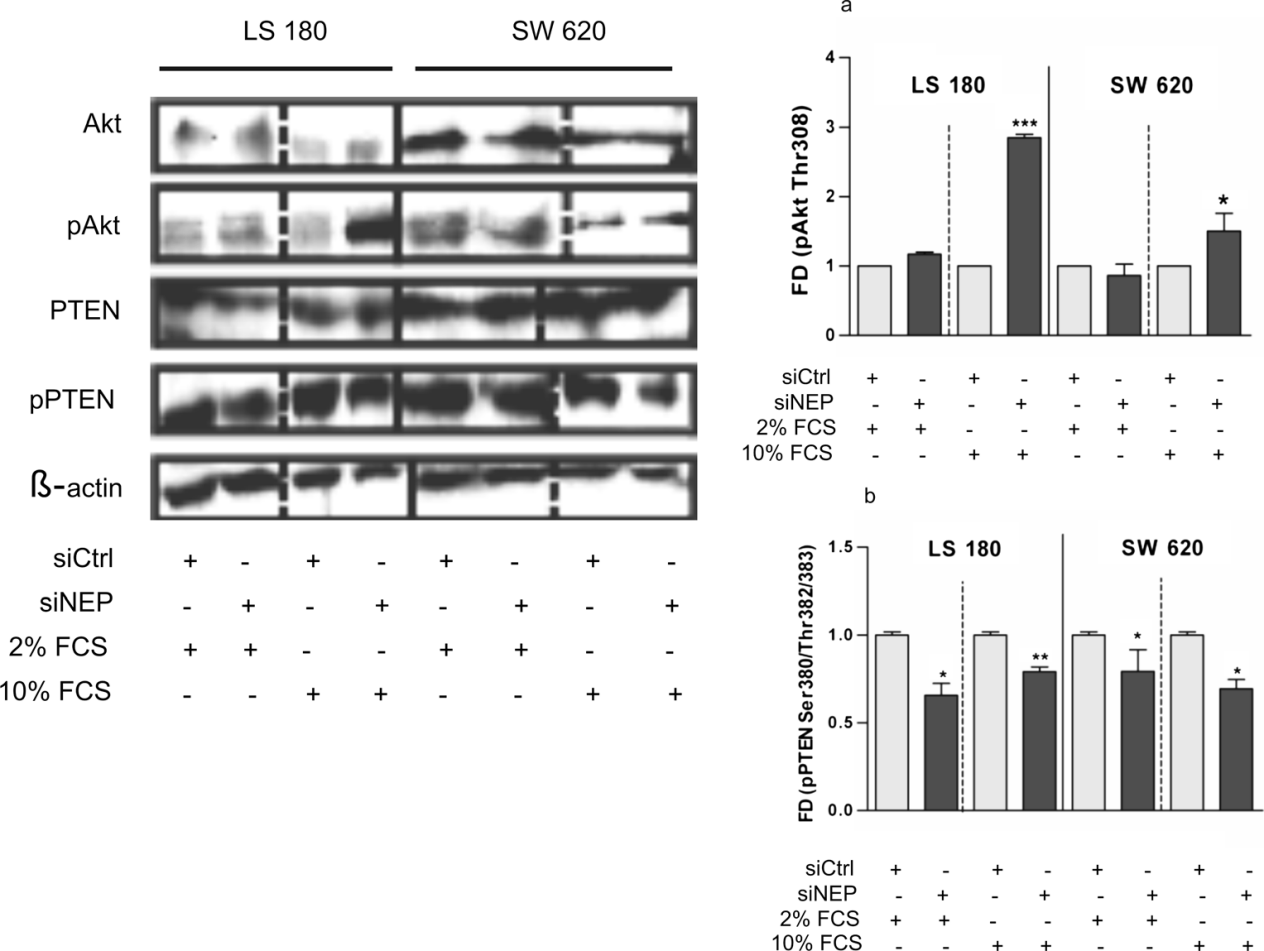
Changes in the phosphorylation of Akt (**a**) and PTEN (**b**) in the LS 180 and SW 620 cells 48 h after NEP silencing. Cells were cultivated in the presence of either 2 % FCS or 10 % FCS. Equal amounts of cell extracts were immunoblotted with antibodies as indicated in the figure. Anti-β-actin antibodies were used as load control. The *left panel* presents representative blots and *graphs* show a densitometric analysis of the bands. *FD*—fold of difference after normalization against β-actin and in comparison with siCtrl. The densitometric analysis was performed on the basis of three independent experiments, and the results are shown as mean values (±SD). Differences were considered statistically significant at *p* < 0.05 (Student’s *t* test) in comparison with siCtrl

**Fig. 11:**
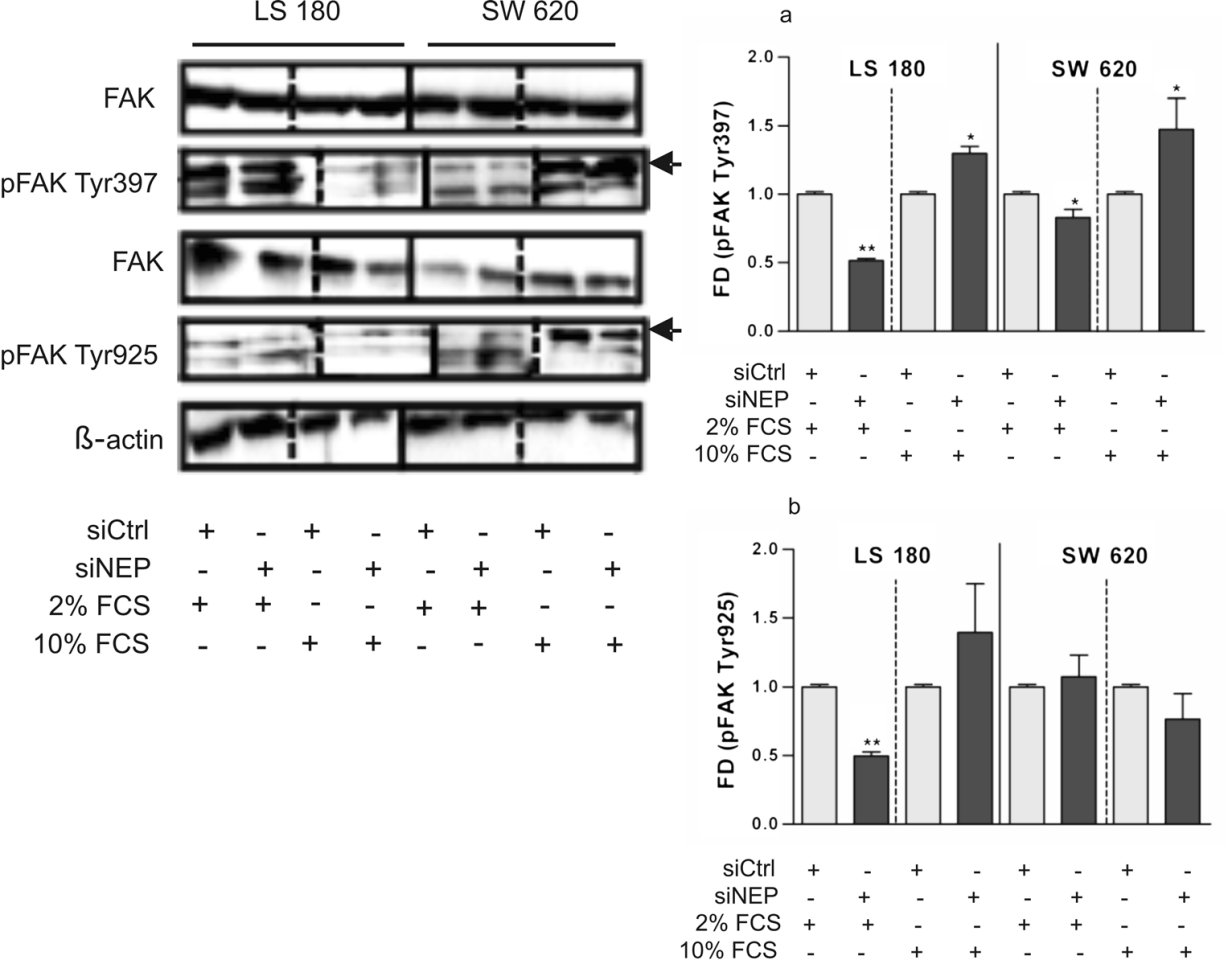
Changes in the phosphorylation of FAK at Tyr397 (**a**) and Tyr925 (**b**) in the LS 180 and SW 620 cells 48 h after NEP silencing. Cells were cultivated in the presence of either 2 % FCS or 10 % FCS. Equal amounts of cell extracts were immunoblotted with antibodies as indicated in the figure. Anti-β-actin antibodies were used as a load control. The *left panel* shows representative blots and *graphs* present a densitometric analysis of the bands. FD—fold of difference after normalization against β-actin and in comparison with siCtrl. The densitometric analysis was performed on the basis of three independent experiments, and the results are shown as mean values (±SD). Differences were considered statistically significant at p < 0.05 (Student’s *t* test) in comparison with siCtrl

Akt/PKB and PTEN are parts of opposite-acting signaling pathways that regulate cell growth and survival. In tumor cells, including colon cancer cells, they are also responsible for the migration and invasive activity, similarly to FAK [7, 8, 12, 22–28]. In this study, it was examined whether these signaling pathways are mediated by NEP. The activity of Akt, PTEN, and FAK was established by western blot analysis on the basis of the level of phosphorylation of these proteins at specific sites. The results were expressed as fold of differences (FDs) in the phosphorylation level after NEP silencing (siNEP) and cultivation of cell cultures in a medium with 2 % (restricted proliferation of the cells) or 10 % (extensively proliferating cells) FCS for 48 h in comparison to a control (1.0) (siCtrl). The results were normalized to the β-actin level. The differences in the phosphorylation level of each protein in the non-siRNA control and siCtrl were not statistically significant. The densitometric analysis revealed that NEP silencing did not alter the expression level of the examined proteins or did not result in their degradation (total amount) but affected their phosphorylation status, and hence, their activity and stability. First and foremost, the results indicated that in comparison to the control, NEP silencing was followed by a statistically significant Akt activation in LS 180 (FD 2.85) and, to some degree, in SW 620 (FD 1.5) cultivated in the presence of 10 % FCS. By contrast, a low concentration of serum did not significantly change the phosphorylation level of Akt (Fig. 10a). In both of the examined cell lines, the increase in Akt phosphorylation was accompanied by a strong, statistically significant decrease in PTEN phosphorylation (LS 180–FD 0.79; SW 620–FD 0.69). Opposite to Akt, PTEN exhibited a lowered phosphorylation level in NEP-silenced cells cultivated in the 2 % FCS medium (LS 180–FD 0.66; SW 620–FD 0.79) (Fig. 10b). NEP silencing resulted in the differential patterns of FAK phosphorylation at Tyr397 and 925 depending on the serum concentration in the medium and the cell line used (Fig. 11a, b). Significantly decreased phosphorylation (FD 0.5 and 0.83) was detected at Tyr397 in the LS 180 and SW 620 cell lines cultivated in the medium supplemented with 2 % FCS. By contrast, the presence of 10 % FCS significantly increased phosphorylation in both cell lines (LS 180–FD 1.3; SW 620–FD 1.48) (Fig. 11a). NEP silencing, exerted a significant effect on the phosphorylation status of FAK at Tyr925 only in the LS 180 cell line. The effect was negative for the LS 180 cells cultivated in the low serum-concentration medium, in comparison to the control (FD 0.5), and positive for cells grown in the medium with 10 % FCS (FD 1.4). In the case of SW 620 cells, NEP silencing did not cause any significant changes in the phosphorylation of FAK at Tyr925 (Fig. 11b).

## Discussion

Colon cancer accounts for a significant part of human malignancies. It is one of the most frequent causes of cancer deaths in Western countries. Surgical resections remain the optimal and often the only therapeutic option for this type of neoplasia, with pharmacological treatments being far from satisfactory. For those reasons, many research groups are prompted to explore the mechanisms of colorectal neoplasia and metastasis. One of the most widely explored problems is the role of proteases and peptidases of different origins, e.g., tumor- and stroma-associated, in the development of malignancy and metastasis. Few of these studies, however, implicate NEP, a cell membrane-bound peptidase, in colon cancer development and metastasis. Most reports evaluate only the relationship between the expression level of the peptidase and the stage/grade or metastasis development of CC in vivo [15–21]. There are some reports which implicate NEP in colon cancer metastasis to the liver but not in biological functions of primary and secondary tumors developing in other sites, e.g., lymph nodes [13, 14]. Given this knowledge, it seemed worth to investigate the role of tumor-associated NEP in the biology of colon cancer cells derived from diverse stages of tumor development (LS180–Duke’s stage B, grade II and SW 620–Duke’s stage C, grade IV) on the cellular and molecular level. To achieve this aim, we used colon cancer cells with inhibited NEP gene expression obtained by RNA interference with siRNA against human NEP. The use of the silencing procedure enabled us to investigate the role of NEP as both a peptidase capable of degrading bioactive molecules and a receptor anchored in the membrane through GPI complexes.

In the present study, we evaluated the presence (immunostaining) and expression level (flow cytometry) of NEP in colon cancer cells originating from different stages and grades of tumor. The immunostaining pattern was consistent with the flow cytometry results and indicated that LS180–Duke’s stage B, grade II, and SW 620–Duke’s stage C, grade IV, exhibited the highest levels of NEP compared to the other colon cancer cell lines examined (HT-29–Duke’s stage A, grade I and SW 948–Duke’s stage C, grade III). A microscopic analysis of the colon cancer cell lines revealed a peripheral (membrane and cytoplasm) localization of NEP. Moreover, flow cytometry showed that MFI was higher in permeabilized cells (data not presented), which could also point to the cytoplasmic localization of NEP. The same type of NEP localization has been reported by Jang et al. [29]. Some results reported in the literature indicate that the expression of NEP is higher in well- and/or moderately differentiated colon carcinomas but not in poorly (metastatic) differentiated ones [16, 18]. On the other hand, there is evidence that the NEP expression level is significantly higher in invasive colon cancer tissue samples (lymphatic, vascular, perineural, and liver invasion) than in lower-stage and non-neoplastic specimens [18, 21, 29]. This difference may result from the fact that in our study, cell lines and not tissue samples were analyzed. Cell lines, due to being cultured for some time, do not necessarily reflect parental tumor. But, considering our results, we can state that there was no correlation between the CD10 expression and the grade of cancer development. Further experiments were performed using cell lines which exhibited the highest level of NEP expression (LS 180 and SW 620). NEP silencing by siRNA in these cell lines resulted in a significant decrease in the expression of this protein, as indicated by immunofluorescence staining and flow cytometry analysis. The number of LS 180 cells with silenced NEP was reduced by about 58 % (MFI reduced by 42 %) and the number of SW 620 cells by about 50 % (MFI reduced by 32 %).

The involvement of NEP in cell survival, growth, and motility has been widely reported for non-neoplastic cells and pancreatic tumor. In the case of the latter, the action of the peptidase was coordinated with the PI3-K/Akt, PTEN, Src, and FAK signaling pathways [2, 6]. There are also studies showing that these signaling pathways mediate the function of colon cancer cells [22–28]. Surprisingly, there are no reports which would implicate NEP in the biology of colon cancer cells in connection with the mentioned signaling pathways. This prompted us to investigate the influence of NEP silencing on colon cancer cell growth, proliferation, and motility. Moreover, we examined whether these processes were mediated by the Akt, PTEN, and FAK signaling pathways. FAK is a non-receptor kinase which, after activation, is autophosphorylated at Tyr397. This enables its binding of diverse signaling molecules, among others Src or PI3K. As a result of these interactions, FAK is phosphorylated at other sites, e.g., Tyr925, what leads to its full activation and regulation of the viability, proliferation, and motile activity of the cells [12].

In preliminary experiments, we determined, for the first time, that NEP is essential for the survival and growth of LS 180 (Duke’s stage B, grade II) and SW 620 (Duke’s stage C, grade IV) colon cancer cells. Peptidase silencing was followed by a statistically significant decrease in the viability and proliferation of these cells. Further studies indicated, for the first time, that NEP silencing resulted in apoptosis of both types of cells when they were cultured in the presence of 2 % (viability assay) or 10 % (proliferation assay) FCS. Moreover, our study is the first to observe that NEP silencing leads to cell cycle arrest in colon cancer cell lines. The arrest at G2/M phase was observed only in the LS 180 cell culture under these culture conditions. Studies on the involvement of NEP in colon cancer survival and growth are scanty. The only report addressing this problem is that by Luo et al., who found that growth of HT-29 cells was inhibited after treatment with antisense phosphorothioate oligodeoxynucleic acid (S-ODN) for CD10 [14].

Further studies on Akt, PTEN, and FAK activity using Western blot analysis revealed that cultivation of NEP-silenced colon cancer cells in the medium with 2 % (restricted proliferation of cells) or 10 % (extensively proliferating cells) FCS resulted in the diverse phosphorylation statutes of these signaling molecules. This is probably caused by different levels of growth factors in the medium. First, the cultivation of cells in the medium with 2 % did not change the phosphorylation level of Akt but decreased the phosphorylation levels of PTEN and FAK (both at Tyr397 and Tyr925). According to some reports, the decrease in PTEN phosphorylation should have resulted in an increase in its activity and its rapid degradation, but only in the membrane-associated form [8, 30]. No such effects were observed in our study. The decreased phosphorylation of PTEN and FAK in NEP-silenced colon cancer cells observed in our study may partially explain the unchanged activity of Akt. However, the mechanism of inhibition of FAK phosphorylation in NEP-silenced colon cancer cells needs to be further investigated. One clue for future studies is the suggestion that NEP-mediated regulation of FAK and Akt activity involves PI3K and Src [6, 12, 22]. In contrast to cultures done with 2 % FCS, cultivation of NEP-silenced colon cancer cells in the medium with 10 % FCS resulted in a significantly increased phosphorylation of Akt and FAK at Tyr397 accompanied by the inhibition of PTEN. These results are consistent with known mechanisms of crosstalk between these signaling molecules. Even so, the activity levels of Akt, PTEN, and FAK obtained in our study do not fully explain the observed inhibition of survival and proliferation of cancer cells, the induction of their apoptosis, and cell cycle arrest. It seems that other signaling pathways may contribute to these effects. One possibility is that the NEP-mediated survival and proliferation of colon cancer cells involves crosstalk among the FAK, Src, and MAPK pathways. A decreased viability of (among others) LS 180 and SW 620 accompanied by inhibited autophosphorylation of FAK (Tyr397) has been examined by Heffler et al. [22]. Moreover, some researchers have reported the induction of apoptosis in colon cancer cells with inhibited FAK (Tyr397), Src, and Akt activities [22, 31]. The decreased viability and induction of apoptosis accompanied by inhibited FAK autophosphorylation is consistent with our results for LS 180 and SW 620 cells cultivated in the medium with 2 % FCS. The inhibition of cancer cell survival and proliferation, induction of apoptosis, and cell cycle arrest at G2/M observed in our experiments may have also been an outcome of the activation of the p38 MAPK pathway. It is known that activated FAK is autophosphorylated at Tyr397 [32]. This enables Src binding and subsequent phosphorylation of FAK by this kinase (among others) at Tyr925. pTyr925FAK interacts with diverse signaling molecules such as Grb2, which leads to the activation of the Ras-ERK signaling pathway and stimulation of proliferation and cell cycle progress [33]. In our study, we observed a decreased or an insignificantly changed phosphorylation level of FAK at Tyr925, which was not coordinated with FAK phosphorylation status at Tyr397. These changes may have been a consequence of disturbed Src/FAK interactions. Moreover, the changes we observed in the phosphorylation status of FAK at Tyr925 may have caused inhibition of ERK activation and a resultant activation of p38 MAPK pathway. These processes could have led to the induction of apoptosis and disturbances in the cell cycle [34, 35]. This hypothesis is supported by Luo et al.’s finding that treatment of HT-29 cells with CD10 antisense S-ODN decrease phosphorylation of ERK1/2 and increased that of p38 MAPK [14].

The motility of cancer cells through the extracellular matrix or the basement membrane plays an essential role in tumorigenesis. Cancer cells can move spontaneously or toward a chemoattractant gradient. Both types of movement enable metastasis of the primary tumor. In our study, NEP silencing resulted in an increased (chemoattracted) migration of both the LS 180 and SW 620 cells. This was accompanied by an increased phosphorylation of FAK at Tyr397 and Akt. The involvement of FAK as a downstream target of Akt in colon cancer cell migration has been investigated by Turečkova et al. [28]. It seems likely that NEP silencing in LS 180 and SW 620 led to an enhanced activity of PI3-K and subsequently Akt. This resulted in FAK activation and increased migration.

Unexpectedly, the present study on the invasiveness activity of colon cancer cells derived from primary tumor (LS 180) and metastatic lymph nodes (SW 620) revealed opposite roles of NEP in these two cell lines. While NEP-suppressed LS 180 cells exhibited enhanced invasiveness through BME, both spontaneous and toward a chemoattractant gradient, SW 620 cells showed decreased invasiveness under the same conditions. We supposed that this disparity was probably due to the different origins of the two cell lines. The different phenotypes of LS 180 and SW 620 cells enable them to create an appropriate microenvironment. At this stage of studies, we cannot explain the exact reason of these results. These issues require further investigation.

In summary, our studies indicate that NEP is involved in colon cancer cell growth, proliferation, and migration/invasiveness, independent of the stage/grade of tumor development. Moreover, we have shown for the first time that NEP mediates motile activities of colon cancer cells through Akt/FAK signaling pathways but does not affect cell growth and survival.
